# Mentes Positivas en Acción: Feasibility Study of a Promotor-Delivered Cognitive Behavioral Stress Management Program for Low-Income Spanish-Speaking Latinas

**DOI:** 10.1089/heq.2019.0012

**Published:** 2019-04-26

**Authors:** Rosa María Sternberg, Anna María Nápoles, Steven Gregorich, Anita L. Stewart

**Affiliations:** ^1^ Center for Aging in Diverse Communities, University of California San Francisco (UCSF), San Francisco, California.; ^2^ School of Nursing, University of California San Francisco (UCSF), San Francisco, California.; ^3^ Intramural Research Program, National Institute on Minority Health and Health Disparities, National Institutes of Health, Bethesda, Maryland.

**Keywords:** Latino stress, promotores, community interventions, mental health care accesss

## Abstract

**Purpose:** Low-income Latino immigrants lack access to mental health providers. We explored the feasibility of training promotores to deliver a stress management program in community settings.

**Methods:** We trained promotores to deliver an 8-week intervention program comprising evidence-based cognitive-behavioral stress management techniques. Trained promotores then delivered the program to Spanish-speaking Latino immigrants.

**Results:** Promotores (*n*=10) improved their knowledge significantly after the training (*p*<0.001) and delivered the program demonstrating excellent fidelity. Participants who received the program (*n*=50) had significantly improved scores on immigration stress, perceived stress, and depressive symptoms (*p*<0.001).

**Conclusion:** It is feasible to train Latino promotores to deliver an effective stress management program to low-income Latino immigrants in their communities. Results contribute to a growing literature on the value of such interventions in community settings. If it is found to be effective in future studies, the program could help fill a large need in the Latino community.

## Introduction

Latinos have high rates of depression^1^ and Latino immigrants are a high-risk group for depression, anxiety, and substance abuse due to unique stressors that include traumatic exposures to violence in their homelands, separation from family, poverty, low levels of education, limited English proficiency, and inferior job skills.^2,3^

In 2014, the United States was home to almost 42.2 million immigrants (13.2% of the total U.S. population), 51.6% of whom were of Latin American origin (21.8 million).^4^ Anti-immigration policies contribute to stress and to a myriad of psychosocial problems for immigrant Latinos and their families.^5–8^ Latinos worry about deportation either for themselves, family members, or close friends.^7,8^ Also, perceived housing and job discrimination are major concerns.^8,9^

Despite their elevated risk of depression, the Surgeon General's office found that Latinos with diagnosable mental disorders underutilize mental health care.^10^ Individual-level factors that may help explain Latino immigrants’ lower mental health service utilization rates include economic and language barriers, lack of mental health insurance, lack of knowledge about where to obtain mental health services, and stigma related to reporting and seeking help for stress and depression-related symptoms.^3,11–13^

Their lower rates of use of services are also related to several system-level factors, such as lack of ethnically diverse and Spanish-speaking mental health providers, providers’ biases and stereotyping, and lack of culturally competent mental health services.^10,14,15^ One approach to increasing access to mental health services is to design interventions that can be delivered in community settings and that are culturally appropriate and in Spanish.^16^ Promotores (community health workers) are being increasingly used to deliver community-based education and interventions, such as healthy lifestyles, with positive results among communities suffering health disparities.^17–21^

In Latino immigrant communities, trained promotores, as trusted community members, can reduce language and cultural barriers because they share the same language, culture, and social contexts of clients or community members. Promotores can easily establish rapport and transmit self-care knowledge and model desired behaviors. However, the participation of promotores in the delivery of mental health programs is not well understood.^22^

The purpose of this article is to describe the results of a feasibility study in which promotores were trained to deliver an intervention called Mentes Positivas en Acción (Positive Minds in Action) created to teach stress management skills to Spanish-speaking Latino immigrants. We aimed to develop a program that could be delivered in local communities to increase the likelihood of participation of this vulnerable group. We first trained promotores in the content and delivery of the program, and they then delivered the program to Latino immigrants in several community settings.

## Methods

### Community partnership

The feasibility study was conducted in Concord, California in 2015. We sought to partner with a community-based organization that already served the Latino immigrant population and could sustain the program if successful. Monument Impact is a community-based organization located in Concord, California that primarily serves disadvantaged, underserved residents by providing programs and services to facilitate their becoming civically engaged, economically self-sufficient, healthy, and safe. Monument Impact already had a promotores model for service delivery and was interested in adding Mentes Positivas en Acción to their set of programs, thus a partnership was established.

### Intervention

The Mentes Positivas program is a group-based stress-management program designed to manage stress and reduce depressive symptoms in lower income, Spanish-speaking Latino immigrants. The intervention draws from three evidence-based programs designed to teach Latinos skills for stress management and prevent and manage depressive symptoms. The programs included: (1) El Curso de Mamas y Bebés^23^; (2) Nuevo Amanecer^24^, and (3) La Prevención de la Depresión.^25^ Program manuals were obtained in English and Spanish from the programs’ authors.

The resulting Mentes Positivas is a manualized program comprised of eight sessions lasting 2 h each; topics covered during each session are shown in Table 1. Sessions included cognitive and behavioral stress management techniques, methods to gain greater control over one's mood, cognitive reframing, the role of increasing pleasant activities, and social skills training. Each session included a homework assignment (e.g., daily record of mood and how it related to their thoughts), which was reviewed at the beginning of each session.

**Table 1.** Mentes Positivas en Acción Program Content

Session	Content
1	□ Sharing personal stories
	□ What is stress and how can you manage it?
	□ Relaxation techniques
	□ Personal affirmations
2	□ What is depression and how can you manage it?
	□ Use of mood thermometer
	□ How thoughts affect your mood
3	□ How to manage your negative thoughts
	□ Practical techniques such as thought postponement—ABC method to examine accuracy of thoughts
4	□ Review sessions 1, 2, and 3
	□ Practice relaxation techniques
5	□ How activities affect your mood
	□ Exploring activities that you enjoy and how they affect your mood
6	□ A plan to improve your life implementing material from previous sessions
	□ A personal contract and how to use it
7	□ Exploring social activities
	□ Goal setting, contract with self
	□ How to manage negative thoughts
8	□ Review program
	□ How to use materials in the future
	□ Graduation ceremony

Participants received the program in small groups of about six to eight participants per group. Groups were offered at a variety of locations in their community, for maximum convenience for participants who lacked transportation. Locations varied and included the conference room of Monument Impact, a community clinic, an apartment complex clubroom, and a classroom in a collaborating community-based organization.

### Training promotores to deliver intervention

We designed a 14-week training for promotores to deliver the intervention using a multifaceted strategy that included didactic presentation of information on stress and depression, several cognitive-behavioral stress management techniques, and guided practice of those techniques and skills. The training curriculum paralleled the content of the Mentes Positivas program and consisted of weekly sessions lasting 3 h each. Training sessions took place in Monument Impact's conference room and were conducted in Spanish by the Principal Investigator, a bilingual/bicultural doctoral-level registered nurse from Chile.

Ten sessions covered the content of the program and four pertained to program delivery skills. The content-based sessions included cognitive and behavioral stress management techniques, methods to gain greater control over one's mood, cognitive reframing, the role of increasing pleasant activities, and social skills training. The sessions on program delivery methods included training in communication, group facilitation, and presentation skills. For each session, the promotores participated in didactic activities, such as discussions of information included in the manual, and received information and tangible resources (e.g., manual handouts, picture cards, and other support materials) that could be shared with program participants.

Promotores also participated in group discussion about how these principles could be applied to fictitious case studies. We paired the promotores so that they would deliver the program as a paired team. Discussions on techniques for working as a team were discussed and practiced during the last four classes of the training. As the training proceeded, we allowed time for promotores to share their own stories of coping with stress and depressive symptoms; occasionally, this led to slight modifications in the program.

Promotores received incentives, including program materials, a certificate of training completion, and a graduation ceremony. Each promotor (men and women) received a $150 gift card for participating in the training. Promotores received an additional $200 gift card upon completing delivery of the 8-week program to community participants.

To provide ongoing technical assistance and enhance fidelity of delivery, promotores were offered continuing education sessions while they were implementing the program in the community. The promotores met monthly with the trainer and research assistant as a group; these group sessions were used to reinforce skills, share their experiences delivering the program to groups in the community, and obtain feedback from one another and the trainer. Fidelity ratings and observations were used to provide feedback to promotores during these sessions.

### Methods of program delivery and monitoring

Each team of promotores delivered the 8 weekly sessions lasting 2 h each. The trainer and research assistant assessed fidelity of delivery of the intervention through direct observations as the promotores delivered the intervention. Two sessions were observed for each team, chosen randomly.

We assessed several aspects of fidelity using a 16-item structured rating scale that assessed the extent to which the teams accurately delivered the program. Examples of fidelity items include the frequency with which follows manual, explains concepts in words that participants can understand, guides participants to practice cognitive reframing, and emphasizes the importance of the homework. Items were rated from 1 to 4 with 1=not at all, 2=a fair amount, 3=a great deal, and 4=consistently. Scores between 3 and 4 indicate good fidelity. Fidelity ratings and observations were used as feedback given to promotores during the continuing education sessions.

### Participants

Promotores recruitment. The trainer and research assistant, in collaboration with Monument Impact, identified potential promotores. We conducted information sessions at Monument Impact's monthly promotores meetings and presented the training program's objectives and how the intervention was designed to be delivered in Spanish to Latino immigrants in the community. Inclusion criteria for promotores included previous experience as a promotor (man) or promotora (woman) in the community, interest in mental health, and ability to speak, read and write Spanish fluently. After providing written informed consent, promotores completed a questionnaire that included information about their age, education, birthplace, marital status, and language spoken at home.

In addition, a needs assessment of their work schedules, family obligations, and transportation was done, to make the training classes congruent with their availability.

Participant recruitment. Participants were recruited in the community through flyers posted at Monument Impact briefly describing the 8-week program being tested for Spanish-speaking Latinos who were feeling stressed or depressed. Eligibility criteria for participants were age 18 years or older and self-identified as Latino who speaks primarily Spanish. Screening was done in person, in Spanish, at Monument Impact's office by the research assistant. Participants were invited to review the schedule of classes and then assigned to a group. At the first session, the research assistant obtained written informed consent and then participants completed a short demographic questionnaire. Participants received a $100 gift card at the end of the study.

### Data collection and measures

Promotores’ knowledge: We assessed promotores’ pre- and post-training knowledge of the program content. At the beginning of each of the first 10 training sessions, promotores completed a brief multiple-choice test assessing their knowledge of the content of each session (sample questions were: What is depression? What are automatic thoughts?). There were no tests administered during the last four sessions dedicated to teaching back and to practicing, and reinforcing promotores’ presentation and facilitation skills.

At the end of the training program (week 14), a 29-item multiple-choice test was administered to measure promotores’ knowledge of the entire training. A sample question was: Automatic thoughts are: (1) necessary to survive, (2) can be thoughts we are not aware that we have, (3) are thoughts we stop having when we are adults, (4) are thoughts we use when we don't when to think too much [with (2) as the correct answer]. The test was scored as the total number of correct answers (range 0–29).

Participant outcome measures: For this feasibility study, we employed a single group pre–post design to evaluate the potential efficacy of the program on participants’ post-intervention stress and depressive symptoms. The research assistant conducted baseline assessments at the beginning of the first session. Spanish questionnaires were self-administered by most participants. Promotores administered questionnaires to a few low literacy participants who could not read and to two who were visually impaired.

Questionnaires assessed basic demographics (age, years of education, language, family income, and health insurance) and the main outcome measures. The outcome measures were also assessed by the research assistant at the end of the final session.

Self-reported outcome measures were the: (1) Stress of Immigration Scale (SOIS), (2) Perceived Stress Scale-7 (PSS-7), and (3) Patient Health Questionnaire-9 (PHQ-9).

The SOIS is a 21-item measure that assesses five domains of stress related to language, immigrant status, work issues, yearning for family and home country, and cultural dissonance among Latino immigrants residing in the United States.^26^ The SOIS was originally tested with a sample of Mexican immigrant women, and its internal consistency reliability exceeded 0.80 for all subscales. Scale scores range from 1 to 5 with higher scores indicating more stress.

A modified version of the Perceived Stress Scale (PSS)^27^ was used to assess general stress. We modified the PSS based on evidence from several studies among Mexican immigrant women that some items did not meet basic psychometric criteria.^26,28^

The modified scale consisted of the seven negatively worded statements (upset because of something unexpected, unable to control important things; felt nervous and stressed; felt that could not cope with all the things you had to do; being mad and feeling out of control; thinking about unfinished things; and difficulties were piling up so that could not overcome them). Each item is rated from 0 to 4, with 4 indicating high stress; we transformed items to a 1–5 scale, thus the final score ranged from 1 to 5. The 7-item scale demonstrated good reliability in this sample (Cronbach's alpha=0.82).

The PHQ-9 is a 9-item self-report measure that assesses depressive symptoms on a 4-point scale (from 0=not at all to 3=nearly every day).^29^ Total scores range from 0 to 27 with higher scores indicating more depressive symptoms. The Spanish version of the PHQ-9 has been found to have good internal consistency reliability (Cronbach's alpha=0.85).^30^

This study was approved by the University of California San Francisco Institutional Review Board.

### Data analysis

We calculated means and proportions for descriptive variables. We calculated means and standard deviations for each of the variables of stress of immigration, general stress, and depressive symptoms. Using linear mixed models to control for clustering within promotores, we assessed changes in participants’ pre- and post-intervention scores on measures of stress of immigration, general stress, and depressive symptoms.

## Results

Ten promotores were recruited and completed the training. Promotores’ mean age was 48.4 years (standard deviation [SD]=8.8, range 37–65). Half was male. By design, all were fluent in Spanish; one-third was fluent also in English. Most were married (90%) and had children (90%). Seventy percent worked outside the home. The average number of years they had worked as a promotor was 6 (SD=4, range 1–14). Half had some college education. Promotores attendance was excellent; only one person missed one class. Nine promotores went on to deliver the intervention in community settings.

Promotores’ knowledge of stress management and depression (possible range of score 0–29) improved significantly after the training. Before the training, the mean knowledge score was 11.9 (SD=5.1, range 5–20), whereas after the training, the mean was 17.6 (SD=3.9, range 11–22). A linear mixed model comparing pre- and post-training scores demonstrated significant increases in promotores’ knowledge from pre- to post-training (*p*<0.001).

The fidelity of the promotores to the program, based on the ratings direct observation ratings, yielded a mean score of 3.4 (SD=0.5) on a 1–4 scale (higher scores=greater fidelity) indicating excellent fidelity to the program. The range of scores across pairs (and one single person) was 2.6–3.9.

We recruited 50 participants to receive the Mentes Positivas program, all of whom were Latino immigrants (one U.S.-born raised in Mexico). Four were unable to finish the eight sessions due to family commitments (*n*=2) and health issues (*n*=2). Additionally, two missed the last two classes and did not complete the post-program measures.

Participants’ mean age was 46 years; most were married and had children (Table 2). The mean years of schooling was 4 (range 2–6). The annual family income reported by the majority (77%) of participants was <$25,000. Most participants were from Mexico. Attendance was excellent with participants attending over 90% of the eight sessions.

**Table 2.** Mentes Positivas en Acción Participants’ Demographic Characteristics (*n*=44)

Characteristic	*n* (%)^a^
Age, mean (SD); range	45.2 (14.0); 20–80
Gender	
Male	10 (23.3)
Female	33 (76.7)
Country of origin	
Mexico	29 (65.9)
Central America	6 (13.6)
Cuba/Dominican Republic	2 (4.6)
South America	5 (11.3)
United States	1 (2.3)
Other	1 (2.3)
Years of school completed, mean (SD); range	4.2 (1.5); 2–6
Annual family income	
<$10,000	11 (25.0)
$10,000–$14,999	8 (18.2)
$15,000–$19,999	5 (11.4)
$20,000–$24,999	6 (13.6)
>$25,000	8 (18.2)
Refused	6 (13.6)
Marital status	
Single	5 (11.4)
Married or partnered	30 (68.1)
Divorced, separated, or widowed	9 (20.5)

^a^ Unless otherwise indicated, percentages based on nonmissing values; 50 enrolled: 4 dropped out and 2 were not at final session when post-assessment was administered.

SD, standard deviation.

Participants’ scores on all three outcome measures improved significantly after completing the program (Table 3). Participants’ perceived stress scores improved from 2.9 (SD=1.1) at baseline to 2.4 (SD=0.9) post-program (*p*<0.001), a difference of about 0.5 SD. Stress of immigration scores improved from 2.3 (SD=0.9) at baseline to 1.8 (SD=0.9) post-program (*p*<0.001), also a difference of about 0.5 SD. Depressive symptom scores improved from 8.8 (SD=6.9) at baseline to 4.4 (SD=6.0) post-program (*p*<0.001), a difference of about 0.75 SD.

**Table 3.** Mentes Positivas en Acción Participants’ Pre-post Outcomes (*n*=44)

Outcome measure	Mean (SD), observed range		*p*-Value^a^
	Pre-program	Post-program	
Stress of Immigration Scale	2.9 (1.1), 1.0–5	2.4 (0.9), 1.0–4.3	<0.001
Perceived Stress Scale	2.3 (0.9), 0.8–4	1.8 (0.9), 0.1–3.7	<0.001
Patient Health Questionnaire-9	8.8 (6.9), 0–26	4.4 (6.0), 0–26	<0.001

^a^ Using linear mixed models controlling for clustering within promotores.

## Discussion

Through this feasibility study, we demonstrated the ability to train successfully community promotores to deliver a cognitive-behavioral stress management program to vulnerable Latino immigrants in community settings. Promotores were able to follow the manual with excellent fidelity and demonstrated improved knowledge of stress and mood management techniques. In turn, participants who completed the eight-session program delivered by the trained promotores demonstrated significant improvements in their levels of immigration stress, general stress, and depressive symptoms.

Our academic–community partnership was unique because Monument Impact already had a promotores model of delivering services to vulnerable Latinos. We utilized this important community asset to house the project, while building its additional capacity to deliver stress management programs. The process of translating evidence-based programs, such as cognitive-behavioral stress management, involved negotiating important tradeoffs between internal and external validity while maximizing program adoption. For example, in each training class, the importance of adhering to the manual was discussed as well of not adding components such as music or exercise to the classes, as was sometimes suggested by promotores. Negotiations required excellent and respectful communication by partners.

The fact that we were able to recruit and retain participants with very low levels of education and income is noteworthy. This supports our model of partnering with a community organization that already serves this population. By conducting all study procedures in the local community and by holding the group sessions in a variety of community settings, we maximized the convenience of participating in the program and the research study.

As in other studies of promotores, our findings indicate that successful promotores-led programs require identification of appropriate promotores, needs assessment of promotores, a significant amount of training, and monitoring of program fidelity.^19,20^ The community partner and promotores were very motivated to complete the program because they felt a strong need on the part of the community for such interventions. We believe that teams of two promotores were better able to manage the groups and exercise greater accountability to follow the program manual with minimal deviation, than the one person who delivered the program alone (due to attrition of one promotore).

During the ongoing technical assistance provided to promotores while they were delivering the program in the community, promotores expressed great satisfaction with the training and the delivery of the program and reported finding great personal meaning in helping their fellow community members. For example, one expressed how participating in this training improved the way she felt about herself as well as how her family and friends perceived her. For her, renewed self-confidence meant a new and improved relationship with her husband, children, extended family, and friends.

Strengths of this feasibility study include near-perfect retention of promotores and excellent attendance of participants. Promotores demonstrated very good fidelity in delivering the program. Limitations include a small sample, lack of a randomized design, and no follow-up assessment after the program was completed.

Our results show the potential benefits of training promotores to deliver a mental health program to low-income Latino immigrants in community settings. The intervention addressed barriers of the stigma of seeking care for depression and the lack of access to culturally appropriate, Spanish-language mental health services. The high prevalence of stress and depressive symptoms in the Latino immigrants who joined the study is consistent with the literature. If the program is found to be effective in future studies using larger samples and a randomized design, it could help fill the large need for such services in the Latino community and reduce mental health disparities for this growing population.

## Abbreviations Used

PHQ-9: Patient Health Questionnaire-9
PSS: Perceived Stress Scale
SD: standard deviation
SOIS: Stress of Immigration Scale
